# Genomic characterization of *Pantoea anthophila* strain UI705 causing urinary tract infections in China

**DOI:** 10.3389/fcimb.2023.1208473

**Published:** 2023-07-14

**Authors:** Yingmiao Zhang, Yue Fan, Yu Zhan, Hao Wang, Xun Li, Hui Wang, Tian Feng, Lifeng Shi, Jing Wang, Hui Wang, Zhongxin Lu

**Affiliations:** ^1^ Department of Medical Laboratory, The Central Hospital of Wuhan, Tongji Medical College, Huazhong University of Science and Technology, Wuhan, China; ^2^ Department of Medical Laboratory, Shiyan Hospital of Integrated Traditional and Western Medicine, Shiyan, China; ^3^ Cancer Research Institute of Wuhan, The Central Hospital of Wuhan, Tongji Medical College, Huazhong University of Science and Technology, Wuhan, China

**Keywords:** *Pantoea anthophila*, urinary tract infection, 16S rRNA, MALDI-TOF MS, whole genome sequencing (WGS)

## Abstract

**Introduction:**

*Pantoea anthophila* (*P. anthophila*) is a Gram-negative bacterium initially isolated from *Impatiens balsamina* in India. *P. anthophila* has been characterized with low pathogenicity, and no human infections caused by this organism have been reported yet. We report the first case of urinary tract infection caused by *P. anthophila* in a 73-year-old man after bladder cancer surgery.

**Methods:**

The bacterial isolate gained from urine was named UI705 and identified as *P. anthophila* by MALDI-TOF mass spectrometry. The genome sequencing and analysis were performed to further characterize the pathogenesis of the clinical isolate.

**Result and discussion:**

To the best of our knowledge, this is the first report of human infection caused by *P. anthophila* in China. The draft genome sequence of *P. anthophila* UI705 provides a fundamental resource for subsequent investigation of its virulence factors, antibiotic resistance, host–pathogen interactions, and comparative genomics of genus *Pantoea*.

## Introduction

The genus *Pantoea* are Gram-negative, facultatively anaerobic, motile, and non-spore-forming bacteria that are generally isolated from agricultural or clinical sources, such as eucalyptus, *Impatiens balsamina*, maize, and human feces (Brady et al., 2008). At present, the genus comprises 18 species with validly published names in the List of Prokaryotic names with Standing in Nomenclature (LPSN) database (Meier-Kolthoff et al., 2022). Among those species, only a few species have been reported to be associated with human infectious diseases, including *Pantoea agglomerans*, *Pantoea ananatis*, and *Pantoea dispersa* (Cruz et al., 2007; Manoharan et al., 2012; Mehar et al., 2013). *P. anthophila* is a member of the genus *Pantoea*, which was originally isolated from *I. balsamina* in India in the 20th century. *P. anthophila* was once assigned to protein profile group VII in the “*Erwinia herbicola*–*Enterobacter agglomerans* complex” (Beji et al., 1988). Studies by Brady et al. placed this strain into a separate cluster and proposed its current name as *P. anthophila* by using multilocus sequence analyses (MLSAs), amplified fragment length polymorphism (AFLP) analysis, and DNA–DNA hybridization in 2009 (Brady et al., 2009). Upon searching in PubMed, only one study reported that *P. anthophila* is pathogenic to plants and naturally causes soft rot disease and cracking on *Clausena lansium* (wampee) in China (Zhou et al., 2015).

The study on pathogen genome is conducive to a comprehensive understanding of pathogenicity and molecular epidemiological characteristics of pathogens. However, there is currently a small amount of genomic information available on *P. anthophila*, mainly due to its rarity. He et al. have recently reported a complete genome sequence of *P. anthophila* CL1 that was initially isolated from symptomatic tissues of wampee, using both next- and third-generation sequencing technologies (He et al., 2022). The genome sequence of *P. anthophila* UI705 in this study represents the first genome of clinical isolate of this microorganism and may provide a better understanding of its pathogenicity in medical practice.

## Materials and methods

### Case presentation

A 73-year-old male patient with frequent urination after bladder cancer surgery was admitted to our hospital on 4 July 2022. The patient underwent bladder tumor resection and bilateral ureteral stent placement for bladder tumor in the trigone 40 days ago, and postoperative pathology showed high-grade non-invasive urothelial carcinoma. The laboratory tests revealed increased neutrophils and fasting blood glucose, but decreased erythrocyte, hemoglobin, and albumin. Urinalysis revealed a WBC count of 523/μl (normal 0–6/μl), an erythrocyte count of 9/μl, urine protein of 1+, and urine glucose of 2+. After isolation of the purified bacterial strain, the patient was diagnosed with urinary tract infection. The details of the case report are presented in the **Supplementary Material**.

### Bacterial isolation

After admission, the clean-catch urine of the patient was collected prior to antibiotic therapy and plated onto Columbia blood agar and MacConkey agar plates (Guangzhou Dijing Microbial Technology Co., Ltd., Guangzhou, China) at 35°C in the presence of 5% CO_2_. After incubation overnight, bacterial colonies with homogeneous morphology were observed and counted on both agar plates, and the bacterial titer was higher than 10^5^ colony-forming units per milliliter (CFUs/ml).

### MALDI-TOF MS identification

The isolated strain UI705 was identified on matrix-assisted laser desorption ionization/time of flight mass spectrometry (MALDI-TOF MS, Bruker Daltonik GmbH, Germany) platform by direct smear of fresh bacterial colony on the target plate in accordance with the manufacturer’s instructions. The acquired spectra data from tested bacteria were compared with the existing known spectrum by using the MALDI Biotyper 3.1 software (Bruker Daltonik GmbH, Germany).

### Biochemical and antibiotic susceptibility test

All the reagents used for biochemical characterization of strain UI705 were purchased from Hangzhou Microbial Reagent, Co., Ltd (Hangzhou, China). The catalytic activity of catalase was detected using 3% (v/v) H_2_O_2_. The oxidase activity was measured by filter paper soaked with the reagent tetramethyl-p-phenylenediamine.

Enzymatic activity and acid production from various carbohydrates of the strain were determined by bacterial microbiochemical reaction tubes according to the manufacturer’s instructions. Antimicrobial susceptibility test (AST) was performed by the Kirby-Bauer disc diffusion method with antibiotics (Thermo Fisher Scientific, Waltham, MA, USA).

### 16S rRNA analysis

The genomic DNA of purified bacteria was extracted using the TIANamp Bacteria DNA Kit (TIANGEN Biotech, Co., Ltd, Beijing, China) according to the instructions of the manufacturer. 16S rRNA gene sequencing was performed with universal primers (27F: 5′;-AGTTTGATCMTGGCTCAG-3′;; 1492R: 5′;-GGTTACCTTGTTACGACTT-3′;). The amplification was performed on C1000 Thermal Cycler (Bio-Rad Laboratories, Hercules, CA, USA) with the following procedure: denaturation at 95°C for 3 min, 30 cycles of denaturation at 95°C for 30 s, annealing at 58°C for 30 s and elongation at 72°C for 1 min, and a further 10 min of elongation at 72°C. The complete 16S rRNA sequence of the strain UI705 was analyzed with the EzBioCloud Database (Yoon et al., 2017a). A phylogenetic tree was constructed using the neighbor-joining method by MEGA software version 11 (Tamura et al., 2021).

### Genome sequencing and annotation

The draft genome sequencing of the strain UI705 was performed using the Illumina HiSeq platform by generating paired-end libraries (PE 150 bp). Fragmented genomic DNA with an average size of 300 bp was selected for sequencing. The raw data of sequencing were evaluated by FastQC v0.11.2 and cut by Trimmomatic v0.36 (Bolger et al., 2014) to obtain relatively accurate and effective data. The filtered reads were assembled into contigs using SPAdes v3.5.0 (Bankevich et al., 2012), and GapFiller v1.11 (Boetzer and Pirovano, 2012) is used to fill the gap between contigs. The genetic elements were predicted by Prokka v1.10 (Seemann, 2014). The average nucleotide identity was calculated using the OrthoANIu algorithm (Yoon et al., 2017b). The DNA–DNA hybridization (DDH) value between UI705 and its related type strain was analyzed by Genome-to-Genome Distance Calculator 3.0 (Meier-Kolthoff et al., 2022). The prediction of antibiotic resistance, virulence factors, determinants of pathogen–host interaction, and carbohydrate-active enzymes was performed by means of the Comprehensive Antibiotic Resistance Database (CARD) (McArthur et al., 2013), the Virulence Factors of Pathogenic Bacteria (VFDB) (Chen et al., 2016), the Pathogen–Host Interactions (PHI) database (Urban et al., 2015), and the Carbohydrate-Active enZYmes (CAZy) Database (Lombard et al., 2014), respectively. The automatic annotation server of protein sequence such as NCBI non-redundant protein sequences (NR) (https://www.ncbi.nlm.nih.gov/), Protein family (PFAM) (Finn et al., 2016), Swiss-Prot (UniProt-Consortium, 2015), Clusters of Orthologous Groups of proteins (COG) (Tatusov et al., 2000), Conserved Domain Database (CDD) (Marchler-Bauer et al., 2013), and Kyoto Encyclopedia of Genes and Genomes (KEGG) (Kanehisa and Goto, 2000) was applied under the right conditions.

## Results

### Physiological, biochemical, and antibiotic susceptibility characteristics

**Figure 1:**
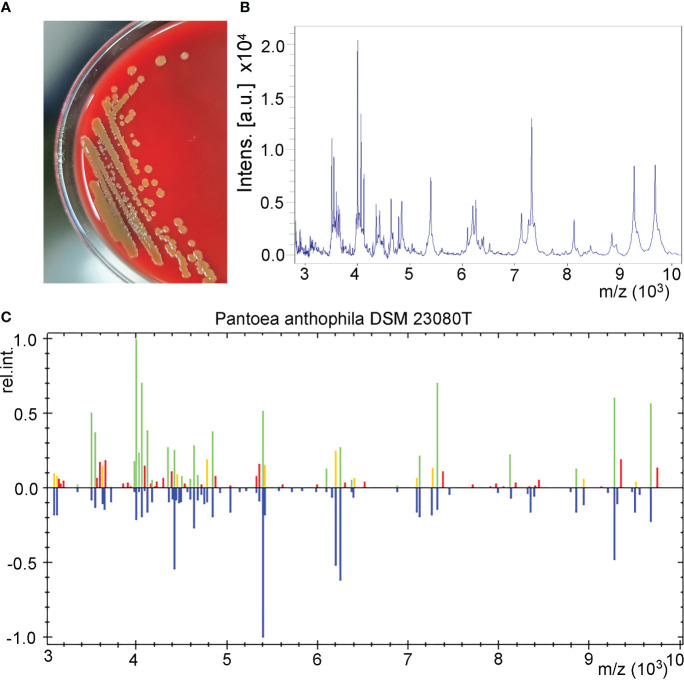
Isolation and identification of *P. anthophila* strain UI705. **(A)** Bacterial colonies on Columbia blood agar after being cultured at 37°C in the presence of 5% CO_2_ for 24 h. **(B)** The spectrogram with protein molecular mass of the strain acquired by MALDI-TOF MS. **(C)** Spectra comparison of the strain UI705 with that of known strains in a database matched to *P. anthophila* DSM 23080T with a high confidence level.

The urine sample developed bacterial colonies on Columbia blood agar after being cultured overnight. The colonies appeared beige to yellow, smooth and circular without apparent hemolytic zones and had diameters between 1 and 2 mm (**Figure 1A**). A spectrogram with protein molecular mass of the strain was acquired by MALDI-TOF MS (**Figure 1B**) and compared with that of known bacterial species in the database provided by the manufacturer. According to the spectra comparison, the strain UI705 was matched to *P. anthophila* DSM 23080^T^ with a high confidence level (**Figure 1C**). Biochemical tests revealed that the strain UI705 is catalase-positive and oxidase-negative. β-galactosidase is produced. Indole, H_2_S, arginine dihydrolase, and urease are not produced. Acid is produced from glucose, mannose, arabinose, rhamnose, and xylose (**Table 1**). The AST via the Kirby–Bauer method showed that the strain is susceptible to almost all tested antibiotics except for ampicillin and cefazolin, which belong to penicillin and first-generation cephalosporin antibiotics, respectively (**Table 2**).

**Table 1:** Biochemical characteristics of strain *P. anthophila* UI705.

Characteristic	Result	Characteristic	Result
Catalase	+	Sorbitol	−
Oxidase	−	Adonitol	−
Glucose	+	Lysine	−
Mannose	+	Ornithine	−
Arabinose	+	Arginine	−
Rhamnose	+	Citrate utilization	+
Sucrose	+	Nitrate reduction	+
Melibiose	−	β-galactosidase	+
Maltose	+	Urease	−
Raffinose	−	Arginine dihydrolase	−
Xylose	+	Motile	+
Aesculin	+	H_2_S production	−

**Table 2:** Antimicrobial susceptibility of *P. anthophila* UI705.

Antibiotics	Content (μg/disc)	Zone of inhibition (mm)	Interpretation
Amikacin	30	24	S
Cefuroxime	30	23	S
Ceftriaxone	30	31	S
Meropenem	10	30	S
Norfloxacin	10	28	S
Cefepime	30	30	S
Tetracycline	30	27	S
Cefazolin	30	14	R
Levofloxacin	5	32	S
Aztreonam	30	31	S
Ceftazidime	30	29	S
Cefoxitin	30	24	S
Sulfamethoxazole	25	31	S
Cefperazone/Sulbactam	105	28	S
Piperacillin/Tazobactam	110	29	S
Ampicillin	10	11	R
Imipenem	10	29	S
Ciprofloxacin	5	30	S
Gentamicin	10	22	S
Fosfomycin	50	28	S

* Drug sensitivity was judged according to EUCAST 2022 standard. S, sensitive; I, intermediate; R, resistant.

### 16S rRNA-based phylogenetic tree

**Figure 2:**
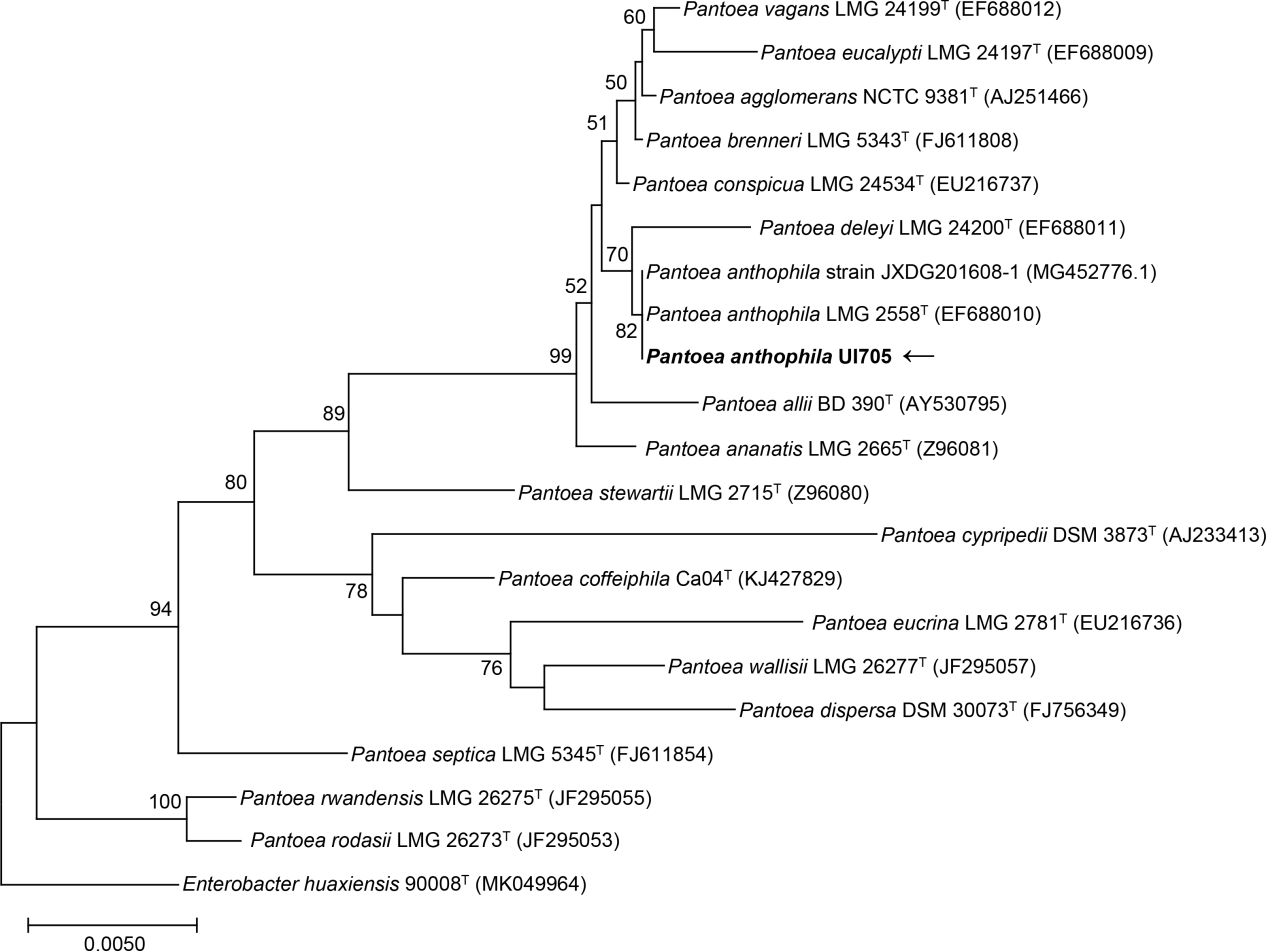
Phylogenetic tree based on the 16S rRNA gene sequences showing the relationship of isolated strain UI705 (black arrow) and members within genus *Pantoea*. The tree was reconstructed by the neighbor-joining method, and *Enterobacter huaxiensis* 90008^T^ (MK049964) was used as an outgroup. Bootstrap values (>50%) based on 1,000 replicates are shown at branch nodes. T, type strain.

To further investigate the phylogenetic features of the isolated strain UI705 in this study, 16S rRNA gene sequencing was performed. The complete 16S rRNA sequence of the strain UI705 was analyzed with the EzBioCloud Database (Yoon et al., 2017a). The strain UI705 exhibited highest (99.64%) 16S rRNA gene sequence similarity with the type strain of *P. anthophila* LMG 2558^T^ (GenBank accession no. EF688010). A total of 1,387 contiguous nucleotides of the strain UI705 were determined and compared with that of *P. anthophila* LMG 2558^T^ with a completeness of 98.5%. Among the 1,387 bases, there was only a five-base difference from strain LMG 2558^T^. The 16S rRNA sequencing results were submitted to GenBank (accession no. OQ704296). Multiple alignments with sequences of the related *Pantoea* and the calculations of the levels of sequence similarity were carried out using the MUSCLE algorithm (Edgar, 2004). A phylogenetic tree was constructed using the neighbor-joining method by MEGA software version 11 (Tamura et al., 2021). The topology of the phylogenetic tree was evaluated by using the bootstrap resampling method with 1,000 replicates. The phylogenetic tree showed that strain UI705 was clustered with the type strain LMG 2558^T^, and this cluster was strongly supported with a bootstrap value of 82% (**Figure 2**). The results of the comparative 16S rRNA gene sequence analysis demonstrated that the isolated strain UI705 belongs to the *P. anthophila* species.

### Whole-genome sequencing of *P. anthophila* UI705

**Figure 3:**
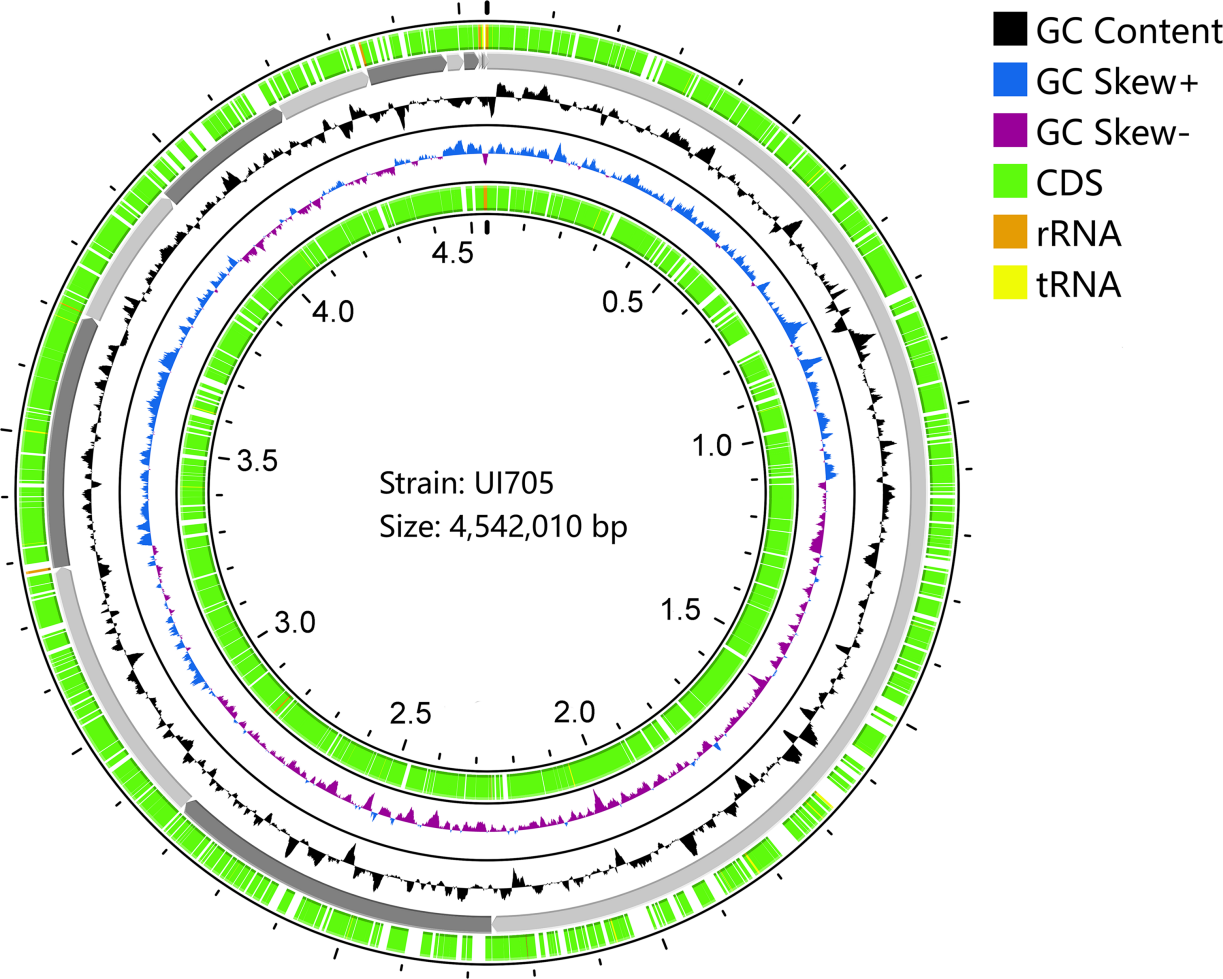
Circular display of the draft genome of *P. anthophila* UI705. The circle from outside to inside represent the following: coding sequences (CDS) on the forward strand (green), the contigs (gray), GC content (black), GC-skew (blue and purple indicate values higher and lower than the mean value, respectively), and CDS on the reverse strand (green). The tRNA (yellow) and rRNA (brown) on both forward and reverse strands are indicated.

**Figure 4:**
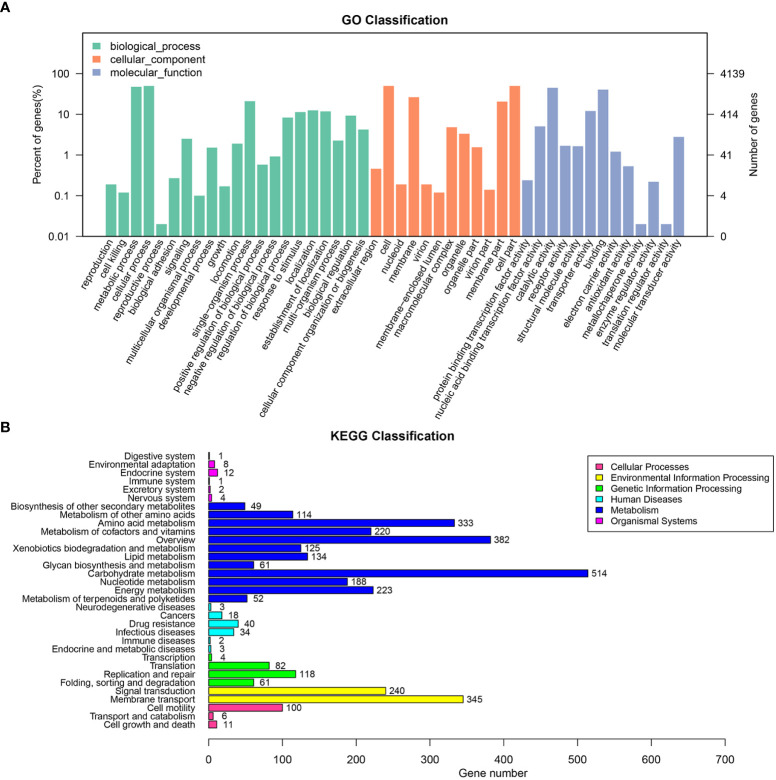
The gene function annotations of the *P. anthophila* strain UI705. **(A)** GO classification describes the properties of genes and gene products as three terms: molecular function (light blue), cellular component (brownish red), and biological process (cyan). The left and right axis indicate percentage of genes and gene numbers, respectively. **(B)** KEGG classification reveals the predicted genes involved in metabolic pathways and human diseases. The numbers on the right of the bar graphs represent predicted genes that are related to correspondent activities.

The draft genome sequence of *P. anthophila* UI705 revealed 4,542,010 bp in size and assembled into 15 contigs, with an N50 value of 564,400 bp and 243.23 × coverage. The GC content of the assembled genome is 56.95% (**Figure 3**), which is consistent with that of other reports available. Automatic annotation with the NCBI PGAP revealed 4,288 protein coding genes. The details of gene element prediction were listed in **Table 3**. Genome-based taxonomy classification using OrthoANIu algorithm validated that UI705 belongs to *P. anthophila* as it revealed a high ANI value of 99.88% between UI705 and the *P. anthophila* type strain LMG 2558^T^. Moreover, the DDH value between UI705 and its related type strain was analyzed by GGDC 3.0 with an estimated value of 99.30% (Formula 2). The gene function annotation of the UI705 was performed in a database that is based on NCBI Blast+ and KEGG Automatic Annotation Server, including CDD, COG, NR, and PFAM, and the number of annotated genes is the highest in the NR database (**Supplement Figure S1**). The assembled sequences of *P. anthophila* UI705 were subjected to GO for further annotation and description of the properties of genes and gene products that were divided into molecular function, cellular component, and biological process (**Figure 4A**). The result from KEGG revealed 100 predictive genes involved in human diseases, mainly those related to infectious diseases and drug resistance (**Figure 4B**), indicating that *P. anthophila* may emerge as a human pathogen.

**Table 3:** Genome features of *P. anthophila* UI705.

Class	Number
Size (base)	4,542,010
G+C content (%)	56.95
Contigs	15
N50 (base)	564,400
Protein coding genes	4,288
Coding ratio (%)	87.6
tRNAs	71
rRNAs	10
Repeat region	0

### Prediction of pathogenicity

**Figure 5:**
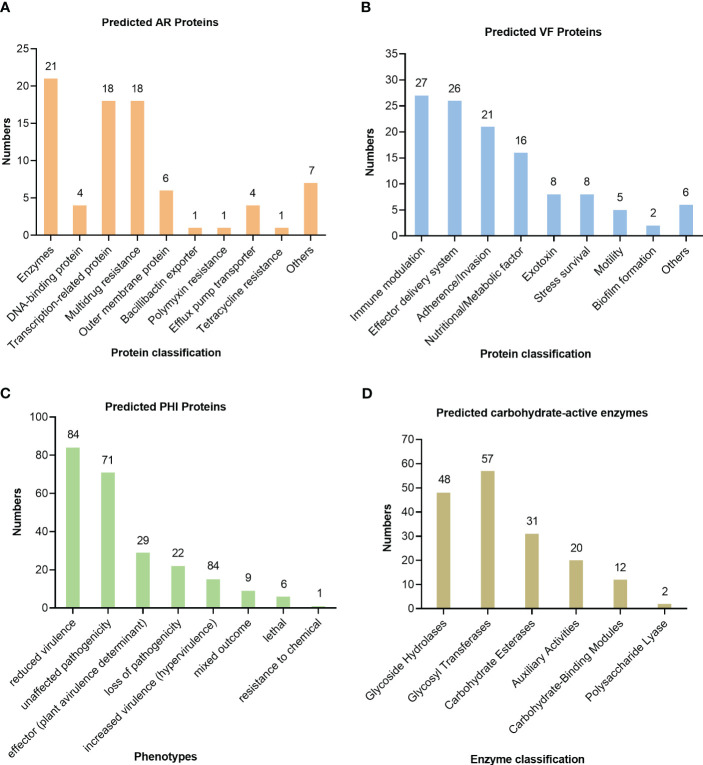
Prediction of pathogenicity of the strain UI705 through the database. **(A)** The antibiotic resistance was predicted by CARD. **(B)** The virulence factors were predicted through the core dataset of the VFDB. **(C)** The pathogen–host interactions were predicted by the PHI database. **(D)** The carbohydrate active enzyme annotation was carried out through the CAZy database. The numbers on the top of the bar graphs represent the predicted proteins in each channel.

The antibiotic resistance of the isolate U1705 was predicted by the CARD and 81 proteins were predicted, such as those related to antibiotic efflux, antibiotic inactivation, and reduced permeability to antibiotic (**Figure 5A**). The antibiotic resistance ontology adeF, rsmA, and CRP were annotated with high percentage identity of matching region (61.44%–89.66%), and the length ratio of reference sequence ranges from 99.24% to 100%. These AROs belong to the resistance-nodulation-cell division (RND) antibiotic efflux pump family and mediate antibiotic resistance in plenty of Gram-negative bacteria (Blair and Piddock, 2009). In addition, a total of 126 virulence factor terms were predicted through the core dataset of the VFDB, which contains 368 predicted proteins (**Figure 5B**). The database predicted the most immune modulation-related proteins, followed by an effector delivery system that includes Type III and VI Secretion Systems (T3SS and T6SS). The T3SS is a needle-like protein complex found in several species of plant or human pathogenic organisms within genus *Pantoea*, contributing to the pathogenicity to their hosts (Correa et al., 2012; Kirzinger et al., 2015). Based on PHI database, the U1705 exhibited pathogenicity to both animals and plants, with a total of 198 PHI-related proteins predicted (**Figure 5C**). There are 11 annotated genes that are related to reduced virulence or loss of pathogenicity in human host. The carbohydrate active enzyme annotation was carried out by CAZy with e-value < 1e−5 and glycosyl transferases remain the highest (**Figure 5D**). The lipopolysaccharide core heptosyltransferase RfaQ and RfaG, and capsular polysaccharide synthesis protein that belong to glycosyl transferases play important roles in the pathogenicity of many bacteria (Aguiniga et al., 2016; St John et al., 2023).

## Discussion

*Pantoea* is a genus of Gram-negative bacteria derived from Erwiniaceae family that was recently proposed as a novel family on the basis of comprehensive and genome-scale taxonomic analysis by Adeolu et al. (2016). The majority of strains within the genus are isolated from plants and the environment, and it is generally considered as a plant pathogen. Among the members of the genus *Pantoea*, only a limited number of species are reported to be associated with human infectious diseases. Currently, the most common causative agent of opportunistic human infections within the genus *Pantoea* is *P. agglomerans*, which can cause infections in different parts of the body, such as bloodstream infections, arthritis or synovitis, periostitis, and endocarditis (Dutkiewicz et al., 2016). Moreover, *P. agglomerans* has caused several nosocomial outbreaks of septicemia since the great epidemic occurred in 1970 and resulted in 152 cases by this organism alone (Dutkiewicz et al., 2016). Infections caused by *P. agglomerans* are mainly from wound infection with plant material and hospital-acquired infection in immunocompromised individuals. Recently, a couple of studies report that *P. dispersa* can cause bacteremia and neonatal sepsis, especially in immunocompromised patients (Mehar et al., 2013; Hagiya and Otsuka, 2014; Asai et al., 2019). Rare cases of bacteriuria are described in *P. ananatis* and *P. stewartii* (De Baere et al., 2004; Cobo et al., 2022). An increasing number of *Pantoea* species related to human infections are described, but the pathogenic mechanisms underlying these species remain unclear.

*P. anthophila* has been characterized with non-pathogenic organism and initially isolated from *I. balsamina* and *Tagetes erecta*, showing no external sign of infection or any negative effect on its hosts (Brady et al., 2009). Wan et al. reported the draft genome sequence of *P. anthophila* strain 11-2 isolated from hypersaline water from the lake on Laysan, Hawaii and numerous virulence-related components were identified based on bioinformatic analysis (Wan et al., 2015). Only one study by Zhou et al. reported for the first time that *P. anthophila* naturally caused soft rot disease and cracking on *C. lansium* (Zhou et al., 2015), indicating that *P. anthophila* is pathogenic to plant.

We encountered the first case of urinary tract infection caused by *P. anthophila* in a 73-year-old man with bladder cancer. The patient denied having been in contact with or being injured by plants or other materials, so the exact source of the isolate from our patient remains unknown. The elderly patient has undergone chemotherapy after surgical treatment of bladder cancer and belongs to the immunocompromised population, which may contribute to the infection of *P. anthophila*. Infections by *Pantoea* species may not present with the conventional symptoms such as fever and leukocytosis, which mainly result from the low virulence of the bacteria. The main symptom of the patient in this study is frequent urination, without fever or other clinical manifestations, and the leukocyte count remained normal with a high percentage of neutrophils (85.6%). Therefore, it is difficult to determine the presence of infection through hematological indicators, and the isolation of pathogenic bacteria from urine confirms the existence of infection. The isolate was susceptible to all antibiotics tested, except for ampicillin and cefazolin, which is consistent with the reported drug sensitivity of *P. stewartia* (Cobo et al., 2022). The patient’s condition improved rapidly due to appropriate antibiotic treatment.

The isolated strain was identified as *P. anthophila* by MALDI-TOF MS, an emerging technology developed in recent years for rapid identification of bacterial pathogens. MALDI-TOF MS can accurately identify commonly isolated micro-organisms due to high coverage rate in the comparison database, but it has limited ability in identification of novel or rare pathogenic species (Ha et al., 2019). Mass spectrometry may be misidentified in the identification of some *Pantoea* species, such as *P. dispersa*, which was initially mistaken as *Klebsiella ozaenae* (Asai et al., 2019). 16S rRNA gene sequencing has been frequently used for molecular classification and phylogeny of bacterial isolates. The 16S rRNA sequence of strain UI705 was found to have a five-base difference from the type strain LMG 2588^T^ within a total of 1,387 nucleotides. A phylogenetic tree also revealed a cluster within the previously reported *P. anthophila* strains and our isolate UI705, indicating that our strain is most closely related to the type strain. Owing to the diversity of microbial species, multi-dimensional analysis, including MALDI-TOF MS and 16S rRNA gene sequencing, will be valuable in accurately distinguishing the large amounts of species from their closest phylogenetic neighbors.

The draft genome sequencing of the clinical isolate UI705 reveals plenty of virulence factors that are involved in human diseases, such as adherence/invasion, toxin, and stress survival. The chaperone protein fimbriae, Myf/pH6 antigen, outer membrane protein OmpU, and multivalent adhesion molecule MAM7 are important adhesins or invasins that mediate the initiation of host–pathogen interactions (Krachler et al., 2011; Liu et al., 2015; Pakharukova et al., 2016). Moreover, there are a number of predicted proteins related to host–pathogen interactions, such as TyrR, yfeAB, Crp, and RelA, and most of the genes are annotated from the reservoirs of *Yersinia pestis* and *Vibrio cholerae*. These results indicate that *P. anthophila* is a potential pathogen in both animal and plant hosts, and further experimental investigations are necessary to determine its pathogenicity. In this case, the *P. anthophila* caused urinary tract infection, which was cured by appropriate treatment with fosfomycin. The antibiotic resistance of the isolate UI705 was characterized by analysis through the CARD database based on the genome with kinds of multidrug resistance proteins. However, the AST results of this study show that the UI705 is only resistant to first-generation cephalosporin antibiotics, which may be due to the poor concordance between bacterial resistance genotypes and phenotypes.

In conclusion, we report the first case of urinary tract infection caused by *P. anthophila* in an elder man with bladder cancer. The clinical isolate was identified and analyzed by means of MALDI-TOF MS, 16S rRNA gene sequencing, and genome sequence. It should be noted that *P. anthophila* may lead to asymptomatic infection in an immunocompromised patient, which deserves the attention of clinicians. Although the pathogenicity of *P. anthophila* is unclear, the present case may indicate an emerging pathogen in medical practice. More cases of infections by this organism should be collected and integrated to explore the potential pathogenicity as well as epidemiology of *P. anthophila* infections.

## Data availability statement

The datasets presented in this study can be found in online repositories. The names of the repository/repositories and accession number(s) can be found in the article/**Supplementary Material**.

## Ethics statement

The studies involving human participants were reviewed and approved by Medical Ethics Committee of the Central Hospital of Wuhan, Tongji Medical College, Huazhong University of Science and Technology. The patients/participants provided their written informed consent to participate in this study.

## Author contributions

YZhang, YF, and ZL designed the research. YZhang, YF, YZhan, and XL performed the experiments. HaW, TF, LS, HaW and JW analyzed the data. YZhang, YF, and HuW wrote the initial draft of the manuscript. All authors contributed to the article and approved the submitted version.
