# Supplementary material for: Genomic characterization of Pantoea anthophila strain UI705 causing urinary tract infections in China
Source: Front Cell Infect Microbiol. 2023 Jul 14;13:1208473. doi: 10.3389/fcimb.2023.1208473 (PMC10375405; doi:10.3389/fcimb.2023.1208473)
Supplement: Supplementary file 1 [file DataSheet_1.docx]

Supplementary Material

Genomic characterization of Pantoea anthophila strain UI705 causing urinary tract infections in China

Yingmiao Zhang^1†^, Yue Fan^1†^, Yu Zhan^1^, Hao Wang^2^, Xun Li^1^, Hui Wang^1^, Tian Feng^1^, Lifeng Shi^1^, Jing Wang^1^, Hui Wang^1^, Zhongxin Lu^1,3*^

*** Correspondence:** Zhongxin Lu, luzhongxin@zxhospital.com.

# Supplementary Data

**Case presentation**

A 73-year-old man with frequent urination after bladder cancer surgery was admitted to our hospital on July 4, 2022. The patient had a history of surgical treatment for gastric cancer and bladder cancer in 2010 and 2017, respectively. laparoscopic cholecystectomy was performed in 2013. The patient underwent bladder tumor resection and bilateral ureteral stent placement for bladder tumor in the trigone 40 days ago, and postoperative pathology showed high-grade non-invasive urothelial carcinoma. The patient was given bladder perfusion with pirarubicin after surgery, and the J tube was removed one month later. Physical examination showed a weak male, with a blood pressure of 144/86 mmHg, a temperature of 36.7 °C, a pulse of 75 beats/min, and a respiratory rate of 18 breaths/min. Laboratory tests showed the following: white blood cell (WBC) count of 9.35×10^9^/L (85.6% neutrophils), an erythrocyte count of 4.03×10^12^/L (normal 4.3-5.8×10^12^/L), hemoglobin of 123 g/L (normal 130-175 g/L), albumin of 37.8 g/L (normal 40-55 g/L), and a fasting blood glucose of 7.48 mmol/L(normal 3.9-6.1 mmol/L. Urinalysis revealed a WBC count of 523/μL (normal 0-6/μL), an erythrocyte count of 9/μL, urine protein of 1+, and urine glucose of 2+. A computerized tomography scan of the urinary tract showed poor bladder filling and slightly thicker bladder wall, indicating the remnant of a previous urothelial carcinoma.

After isolation of the bacterial strain UI705, the patient was diagnosed with urinary tract infection. An antibiotic susceptibility test (AST) via the Kirby–Bauer method showed the strain is susceptible to almost all tested antibiotics except for ampicillin and cefazolin (Table 1). The patient was treated with intravenous fosfomycin (2g/12h) according to the result of AST. On the third day after admission, the patient underwent cystoscopy and transurethral resection of the bladder wall where the previous tumor had been located, reaching deep muscularis. The postoperative pathology examination of bladder neoplasm showed mainly fiber and smooth muscle components, obvious necrosis, more neutrophils and lymphocytes infiltration, and a few free urothelial masses with certain atypia. After three days of anti-infective and supportive treatment, the patient's physiological conditions and inflammatory indicators were normalized, and urine culture was negative. The patient was discharged after one week of hospitalization, and three-month follow-up showed no abnormality.

# Supplementary Figures and Tables

## Supplementary Figures


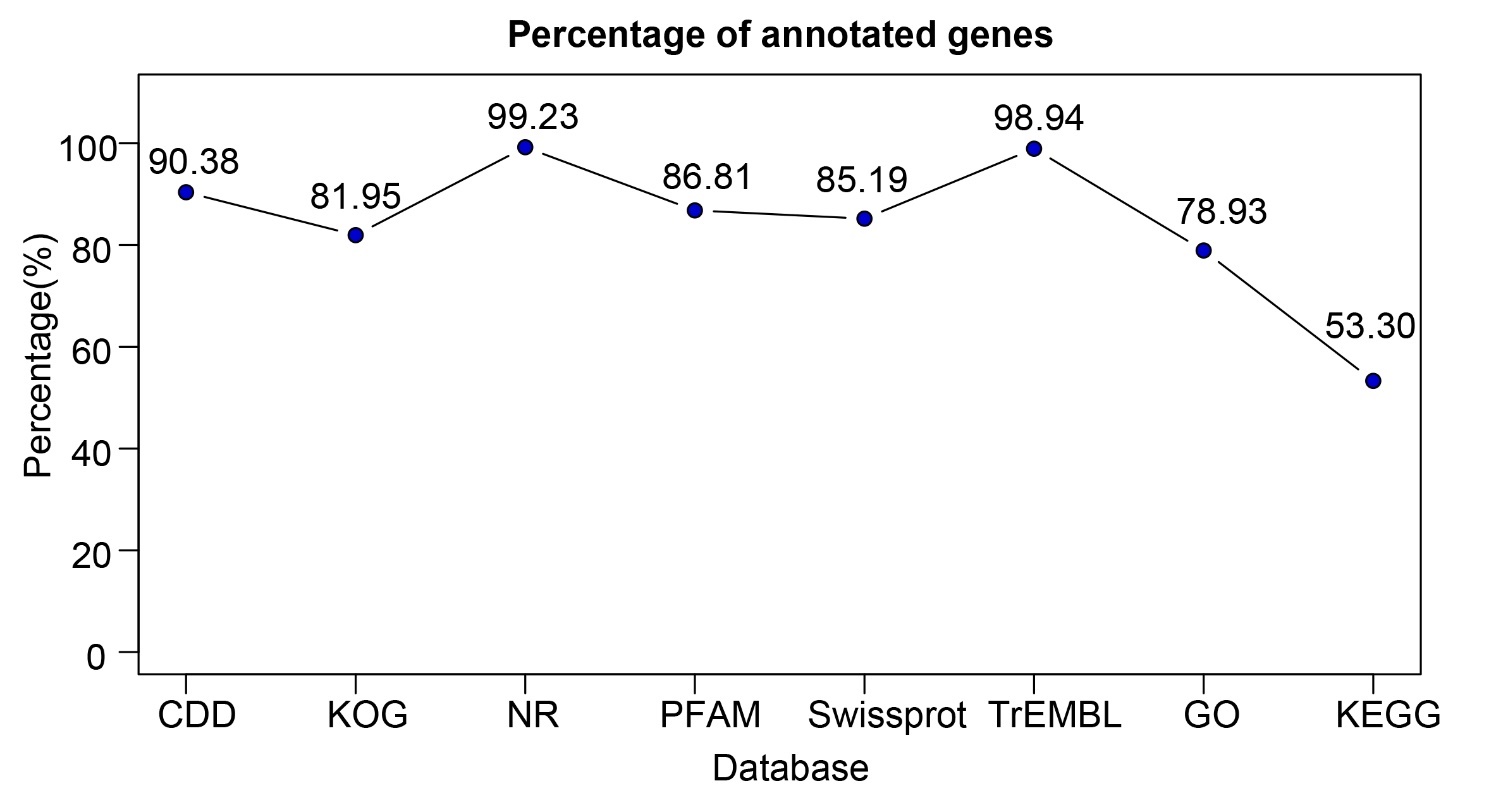


**Supplementary Figure S1.** Percentage of annotated genes of P. anthophila UI705 in different databases. CDD, Conserved Domain Database; COG, Clusters of Orthologous Groups of proteins; NR, NCBI non-redundant protein sequences; PFAM, Protein family; Swissprot, A manually annotated and reviewed protein sequence database; TrEMBL, A supplement to Swissprot; GO, Gene Ontology; KEGG, Kyoto Encyclopedia of Genes and Genomes.
